# Determining the impact of the Zika pandemic on primary care providers’ contraceptive counseling of non-pregnant patients in the US: a mixed methods study

**DOI:** 10.1186/s12913-021-07170-0

**Published:** 2021-11-09

**Authors:** Jennifer E. Kaiser, Eduardo Galindo, Jessica N. Sanders, Rebecca G. Simmons, Lori M. Gawron, Jennifer S. Herrick, Benjamin Brintz, David K. Turok

**Affiliations:** 1 University of Utah, Division of Family Planning, 50 North Medical Drive, Salt Lake City, UT 84132 USA; 2 University of Utah, Study Design and Biostatistics Center, 295 Chipeta Way, Salt Lake City, UT 84122 USA

**Keywords:** Contraception, Counseling, Zika virus, Primary care

## Abstract

**Background:**

Global pandemics like Zika (ZIKV) factor into pregnancy planning and avoidance, yet little is known about how primary care providers (PCPs) incorporate public health guidance into contraceptive counseling. Study objectives include: 1) determining the impact of the ZIKV pandemic on contraceptive counseling changes; and 2) assessing PCP knowledge and practice regarding contraception, ZIKV, and CDC ZIKV guidelines.

**Methods:**

Study components included: (1) a retrospective review of electronic health records of non-pregnant, reproductive age women presenting for preventive health visits between 2014 and 2017 assessed using interrupted time series analyses (ITSA) to identify changes in documentation of ZIKV risk assessment and contraceptive counseling; and (2) a sequential, cross-sectional study with quantitative surveys and qualitative, semi-structured interviews of PCPs providing preventive care to non-pregnant patients at eight federally qualified health centers in Utah. We performed descriptive analyses on survey data and analyzed qualitative data for dominant themes using a modified Health Belief Model.

**Results:**

We conducted 6634 chart reviews yielding 9840 visits. The ITSA did not reveal changes in ZIKV risk assessment or contraceptive counseling. Twenty-two out of 40 (55%) eligible providers participated in the provider component. Participants averaged 69 and 81% correct on contraceptive and ZIKV knowledge questions, respectively. Sixty-five percent reported counseling consistent with CDC ZIKV guidelines. Qualitative analysis found providers unlikely to prioritize ZIKV risk assessment in contraceptive counseling for non-pregnant patients.

**Conclusions:**

PCPs who care for non-pregnant women are knowledgeable about contraception and ZIKV; however, there was no change in ZIKV risk assessment or contraceptive counseling. This stresses the importance of developing strategies to improve guideline uptake.

**Supplementary Information:**

The online version contains supplementary material available at 10.1186/s12913-021-07170-0.

## Background

The 2015 Brazil Zika virus (ZIKV) outbreak and its subsequent spread from South to Central and North America brought a newfound sense of urgency to expand contraceptive counseling and access among populations at risk for ZIKV and unintended pregnancy [1–4]. Epidemiologic research quickly identified the association between ZIKV and birth defects (the ZIKV Congenital Syndrome) and confirmed sexual transmission [5, 6]. In response to this public health crisis, the Centers for Disease Control and Prevention (CDC) issued multiple guidance statements on ZIKV prevention counseling. These guidelines prompted providers to inquire about recent or planned travel to areas with active outbreaks and to counsel on pregnancy avoidance though condom and other contraception use [6–8].

Hispanic/Latinx women residing in the United States may be at increased risk for ZIKV, compared to non-Hispanic/Latinx women, as they are more likely to travel to areas with active ZIKV transmission or have partners that travel to or live in these areas [9]. Hispanic/Latinx women are also at increased risk of unintended pregnancy compared to the entire U.S. population [10]. In Utah, 15% of the population identifies as Hispanic/Latinx, and this increases to 19.9% in Salt Lake County [11]. In addition, 58% of women aged 13 to 44 in Utah report an unmet need for contraceptive services, and 14% of those identify as Hispanic/Latinx [12]. Personalized counseling and adherence to CDC ZIKV guidelines for non-pregnant women are necessary to reduce ZIKV risk in this vulnerable population.

Many non-pregnant women rely on primary care providers (PCPs) for routine and preventive reproductive healthcare. However, we lack information on provider adherence to CDC ZIKV guidelines and assessments of their knowledge or comfort level regarding ZIKV and contraceptive counseling. This study focuses on care provided to non-pregnant women at possible risk of ZIKV residing in Salt Lake County, Utah. We aimed to: 1) determine the impact of ZIKV on changes in contraceptive counseling; and 2) assess PCP knowledge and practice regarding contraception, ZIKV and CDC ZIKV guidelines.

## Methods

We performed this mixed methods study at the Community Health Centers, Inc. (CHC) in Salt Lake County, Utah. Approximately 75% of the 3000 female patients of reproductive age they see per year for preventive health services identify as Hispanic/Latinx. At the time of the qualitative portion of the study, the CHC employed 44 providers including Family Medicine physicians and advanced practice clinicians (APCs). The University of Utah IRB approved all components of this study and determined the study exempt. Participants received an IRB approved consent cover letter prior to enrollment and provided implied consent upon completion of the survey and interview. The CHC Board of Directors gave permission to recruit practitioners and to access charts for review.

### Assessment of ZIKV risk assessment in contraceptive counseling visits

To determine evidence of ZIKV risk assessment in contraceptive counseling visits, we conducted a retrospective review of electronic health records (EHRs) for all preventive health visits at the CHC. The CHC’s EHR, eclinical®, has been in use since 2010. We identified records based on initial and established preventive care CPT codes for adolescents and adults (99,385, 99,395, 99,384, 99,394, 99,386, 99,396). Eligible records for review included any new or established non-pregnant female patient between ages 15-49 who presented for preventive care between January 1, 2014 and December 31, 2017. We excluded patients with documented tubal ligation or hysterectomy.

To determine whether the ZIKV pandemic changed contraceptive counseling rates among non-pregnant patients, we divided our study into a pre-intervention period (P1: January 1, 2014 - December 31, 2014), a six-month transitional lag period including the start of the outbreak in May 2015 where information of the virus was limited but increasing (P2: January 1, 2015- June 30, 2015) and an intervention period (P3: July 1, 2015 - December 31, 2017) when US media attention grew and the CDC issued multiple ZIKV guidelines on pregnancy planning and contraceptive use.

To determine whether ZIKV was incorporated into contraceptive counseling, we defined ZIKV risk assessment as: a) documentation of patient travel to an endemic ZIKV area; OR b) documentation of partner travel to an endemic ZIKV area; OR c) any documentation of ZIKV risk in relation to timing of pregnancy; OR d) any documentation of ZIKV risk in relation to sexual transmission. We determined contraceptive counseling to be present in charts if: a) documentation of a contraceptive method in the Assessment/Plan area, b) documentation of a contraceptive method beyond the history of present illness, or c) documentation that the patient does not desire a method at this time. Other data collected included demographic information and documentation of contraceptive counseling. JK trained a team of seven research assistants (RAs) on data abstraction for the EHR system. When each RA completed 10 charts, JK audited each chart for accuracy and consensus across RAs. We entered chart review data into the University of Utah’s Research Electronic Data Capture (REDCap).

The primary estimates of interest for this portion of the study were to assess the effect of the intervention period on ZIKV risk assessment and contraceptive counseling documentation rates. We do so by modeling both the immediate effect as well as any effect over time during both a 6-month transition period and intervention period. We also conducted a secondary analysis which included a main effect as well as interactions by period for Hispanic/Latinx ethnicity. We implemented the interrupted time-series analysis (ITSA) framework using a generalized linear mixed effects model (GLMM) with a logit link to model each outcome and included the following covariates: time period indicator, a linear effect for date, date by period interactions, ethnicity, ethnicity by date and time period interactions, visit provider gender, visit provider type, and visit number for a patient. The date variable was made numeric with the first date (January 1, 2014) set to 0 and then scaled (subtracted mean and divided by standard deviation). We additionally include a random effect by patient id and a random date slope by provider to account for correlation in outcomes between visits of the same patient, correlation of outcomes of the same provider, and the possibility of different behavior by provider over time. Due to the instability of generalized linear mixed effects models, we were not able to use all random effects described for all models fit. Analyses were conducted in R version 3.6.2 [13].

The minimun number of charts per time period (month) for appropriate power in an ITSA is 50 [14, 15]. We, therefore, aimed to abstract at least 50 charts per month from January 2014 to December 2017.

### Assessment of provider knowledge and practice of ZIKV risk assessment

We performed a sequential, mixed-methods, cross-sectional study comprised of a descriptive quantitative survey and semi-structured, qualitative interviews. Between June 4, 2018 and August 9, 2018, we recruited PCPs, including physicians, nurse practitioners (NPs), and physician assistants (PAs) via convenience sampling through recruitment emails sent individually to each eligible CHC provider. A priori, we excluded three physician participants not board certified in Family Medicine (one obstetrician/gynecologist and two pediatricians), due to small numbers, leaving a total of 40 PCPs for recruitment. After recruiting 15 participants to the quantitative and qualitative arm in 2018, we then optimized quantitative numbers and recruited the remaining 7 survey participants from April 11 to 30, 2019. We included practitioners who perform preventive health visits for women of reproductive age (15 to 49 years). All providers are bilingual in English and Spanish. All of the participants in the survey and interview were active employees of the CHCs during the chart review time period above (January 2014 through December 2017).

### Quantitative survey

We adapted survey questions from the World Health Organization ZIKV survey and contraceptive knowledge questions from a widely-cited study [16, 17]. We created questions on the adherence to CDC ZIKV guidelines de novo, based on the Interim Guidance for Preconception Counseling and Prevention of Sexual Transmission published in September 2016 (accessed June 2018) [6]. This CDC guideline contained recommendations to prevent ZIKV transmission for non-pregnant women. We performed cognitive interviews during survey development and piloted the completed survey with 5 women’s health providers outside the CHC. We emailed the final 37-question survey to participants to complete online. This included 6 contraceptive knowledge questions, 7 ZIKV knowledge questions, and 4 CDC ZIKV guideline adherence-related questions. The remainder of the questions assess demographics and clinical characteristics (Additional file 1). Data collection occurred through the REDCap system. We performed descriptive analyses of the survey data using Stata 15 software (College Station, TX).

### Qualitative interview

After the survey, participants during the initial recruitment phase (June to August 2018) completed a semi-structured, in-depth telephone interview with an experienced qualitative research assistant, EG. He was not previously known by any of the study participants. EG contacted participants to schedule a telephone interview, conduct the interview, and distribute compensation gift codes by email. To avoid bias, principal investigator, JK, did not conduct any interviews as she works clinically with several of the participants. Recruitment emails clearly stated the purpose of the study to evaluate knowledge and practices regarding contraception and ZIKV. We recruited participants into the qualitative arm until we achieved thematic saturation defined as no further major themes arising during the iterative process. We achieved thematic saturation after the first round of recruitment and did not re-open the qualitative arm for additional recruitment in 2019.

**Fig. 1:**
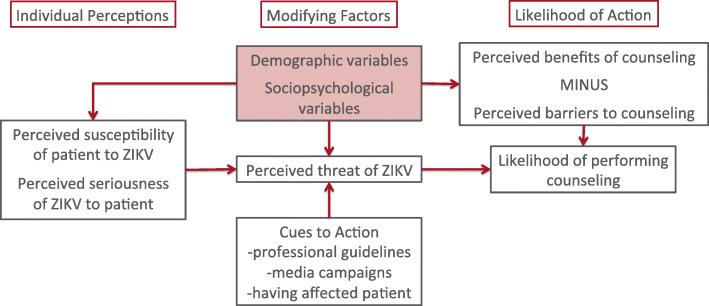
Modified health belief model used to develop the interview guide. Health Belief Model depicting how individual perceptions and modifying factors inform the likelihood of providers counseling their patients about contraceptive use and ZIKV virus. The shaded box will be addressed through quantitative surveys. Adapted from Janz & Baker, 1984 [18].

We developed the semi-structured interview guide using the Health Belief Model as a conceptual framework to specifically address changes in contraception counseling in relation to perception of ZIKV risk (Fig. 1) [18]. We piloted the interview guide with two CHC physicians who were then excluded from formal participation. Examples of questions include: “how serious do you feel the risk of Zika is to non-pregnant patients” and “how useful do you find the CDC guidelines on Zika for non-pregnant women to your everyday practice?” All participants completed one 20-30 min telephone interview in 2018 with only EG present on the line. We audio recorded and professionally transcribed the de-identified interviews. Transcripts were not returned to participants. We uploaded transcripts into Dedoose online software for coding and analysis (version 8.3.35 (2020), Los Angeles, CA).

Using the iterative process, JK and EG developed a list of codes to identify major themes corresponding to the main domains in the Health Belief Model (Fig. 1).

JK and EG jointly reviewed 5 transcripts and discussed major themes. Based on these major themes, they created a codebook. JK then coded 5 interviews independently. EG reviewed the applied codes and we discussed new codes and discrepancies until consensus was achieved. JK and EG each coded half of the remaining interviews and reviewed the others’ codes. A third investigator was available to resolve any discrepancies, but was not needed. Participants did not provide feedback on findings, but findings were presented at a CHC’s staff meeting on January 8th, 2020**.**

## Results

### ITSA results

We abstracted a total of 6634 charts. From these, 9840 visits were included in the analyses, including 6464 unique patients and 56 unique providers. Table 1 provides a descriptive summary of the charts reviewed.

**Table 1:** Descriptive summary of charts reviewed

Variable		Number of charts N(%)
Year	2014	1805 (18.3%)
	2015	2085 (21.2%)
	2016	2827 (28.7%)
	2017	3123 (31.7%)
Contraceptive Counseling present		3888 (39.5%)
ZIKV Risk Assessment present		95 (1%)
Hispanic/Latinx ethnicity, patient		8805 (89.5%)
Number of visits per patient	1	6404 (65.1%)
	2	2315 (23.5%)
	3	782 (7.9%)
	4	237 (2.4%)
	5	72 (0.7%)
	6	30 (0.3%)
Provider Type	Physician	3375 (34.3%)
	NP	1406 (14.3%)
	PA	5059 (51.4%)
Provider gender: Woman		6453 (65.6%)

Descriptive summary of all charts reviewed between January 1, 2014 and December 31, 2017. Contraceptive counseling and ZIKV risk assessment denote number of charts with documentation of these assessments. *N* = 9840. NP = nurse practitioner; PA = physician assistant.

**Fig. 2:**
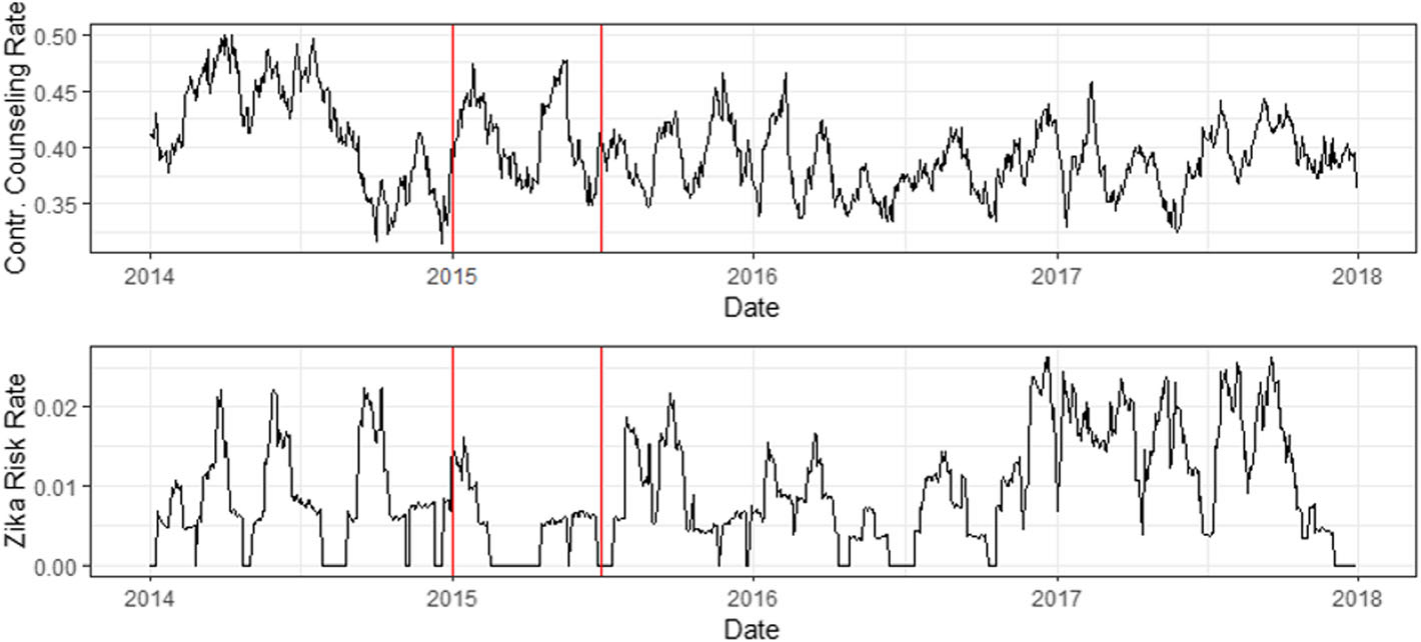
Interrupted time series analysis of contraceptive counseling and ZIKV risk assessment. Trends of both contraceptive counseling and ZIKV risk assessment before and during the ZIKV pandemic. Red lines depict the six-month lag period that accounts for the onset of the pandemic, when clinical guidance was limited

Figure 2 shows the incidence of ZIKV risk assessment and contraceptive counseling in EHRs over the study time period.

Table 2 shows that an increasing number of Visits increases the chance of documentation of contraceptive counseling. Additionally, we have some evidence, although non-significant, that the treatment period has a steeper slope and higher rate of documentation for Hispanic/Latin women than the pre-treatment period.

**Table 2:**
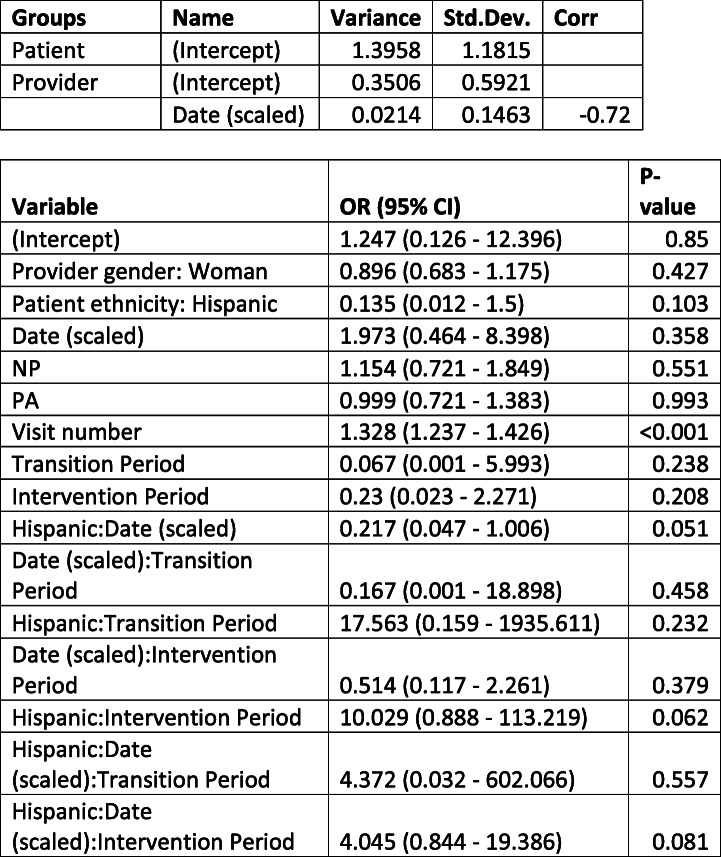
Estimates of change in contraceptive counseling among all patients

Estimates of change in electronic health record documentation of contraceptive counseling before and after the ZIKV pandemic using a generalized linear effects model. Estimates (Odds Ratio) and 95% confidence intervals for all variables in a GLMM (logit link) regressed on the documentation of contraceptive counseling. We additionally present the variance and correlation estimates from the random effects. *NP* nurse practitioner, *PA* physician assistant.

We could only fit a GLMM with random provider intercept for the ZIKV risk outcome. This is likely due to the very low rates of documentation of ZIKV risk and very little variance by patient and providers over time resulting in instability of the model. As a result, we don’t account for any patient correlation other than through the Visit number variable. We fit two models, one which utilized the same periods as above (Table 3), and the other which only included the post-treatment period and looked at Hispanic/Latinx ethnicity (Table 4). Neither showed evidence of a change in rate of zika risk documentation over time or a difference by Hispanic/Latinx ethnicity.

**Table 3:**
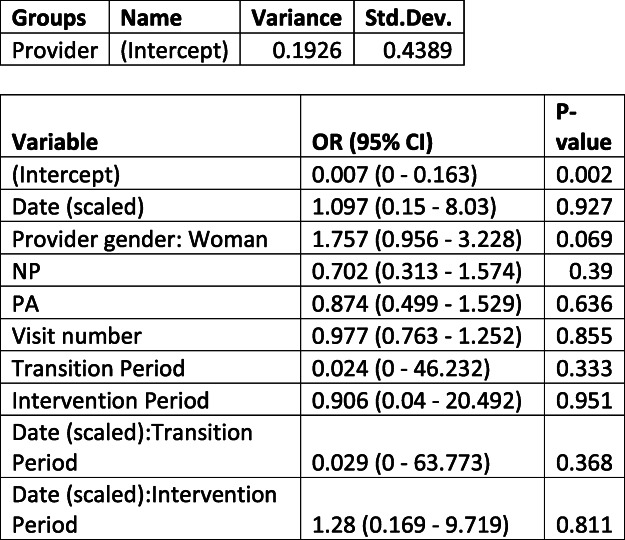
Estimates of change in ZIKV risk assessment among all patients

Estimates of change in electronic health record documentation of ZIKV risk assessment before and after the Zika pandemic using a generalized linear effects model. Estimates (Odds Ratio) and 95% confidence intervals for all variables in a GLMM (logit link) regressed on the documentation of ZIKV risk assessment. We additionally present the variance and correlation estimates from the random effects. *NP* nurse practitioner, *PA* physician assistant.

**Table 4:**
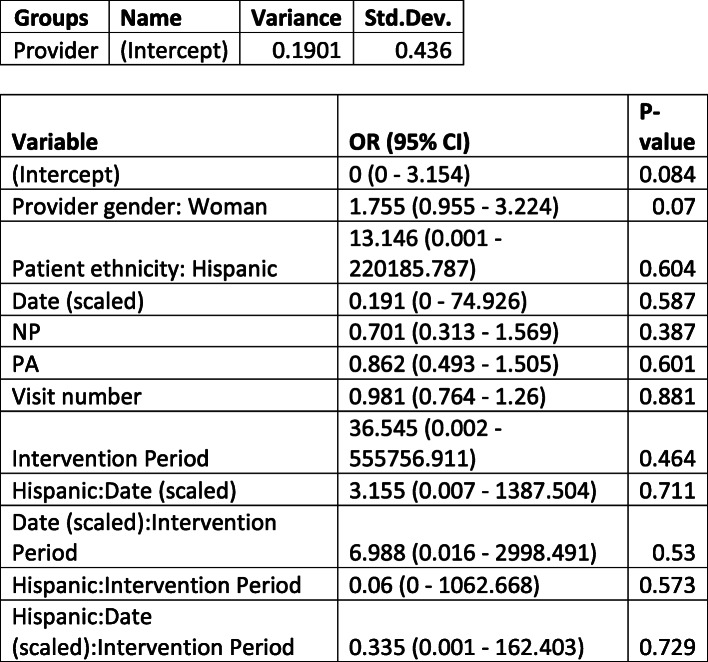
Estimates of change in ZIKV risk assessment documentation among Hispanic/Latinx patients

Estimates of change in electronic health record documentation of ZIKV risk assessment, including covariates to estimate this change for Hispanic/Latinx patients before and after the ZIKV pandemic using a generalized linear effects model. Estimates (Odds Ratio) and 95% confidence intervals for all variables in a GLMM (logit link) regressed on the documentation of ZIKV assessment. We additionally present the variance and correlation estimates from the random effects. *NP* nurse practitioner, *PA* physician assistant.

### Survey results

Twenty-two out of 40 eligible providers participated in the survey (55% response rate). Provider demographics are listed in Table 5.

**Table 5:** Characteristics of providers completing the survey

Characteristic	
Years practicing at the CHC (median, IQR)	5.0 [3.0, 17.0]
Number of women of reproductive age seen in average half day (range)	2-20
Provides reproductive health counseling^a^	
Yes	96%
Age (median, IQR)	39.0 [35.0, 52.0]
Gender (%)	
Woman	55%
Race (%)	
White	96%
Ethnicity (%)	
Not Hispanic/Latino	86%
Provider type (%)	
Physician	55%
Physician Assistant	32%
Nurse Practitioner	14%
Years in practice (median, IQR)	6.0 [4.0, 17.0]
Years in primary care (median, IQR)	6.0 [3.5, 17.0]
Years caring for Hispanic/Latino patients (median, IQR)	5.5 [3.5, 17.0]

Characteristics of providers completing the survey arm. *N* = 22.

^a^I.e. preconception counseling, contraceptive counseling, sexually transmitted disease counseling.

*CHC* Community Health Centers, *IQR* interquartile range

**Fig. 3:**
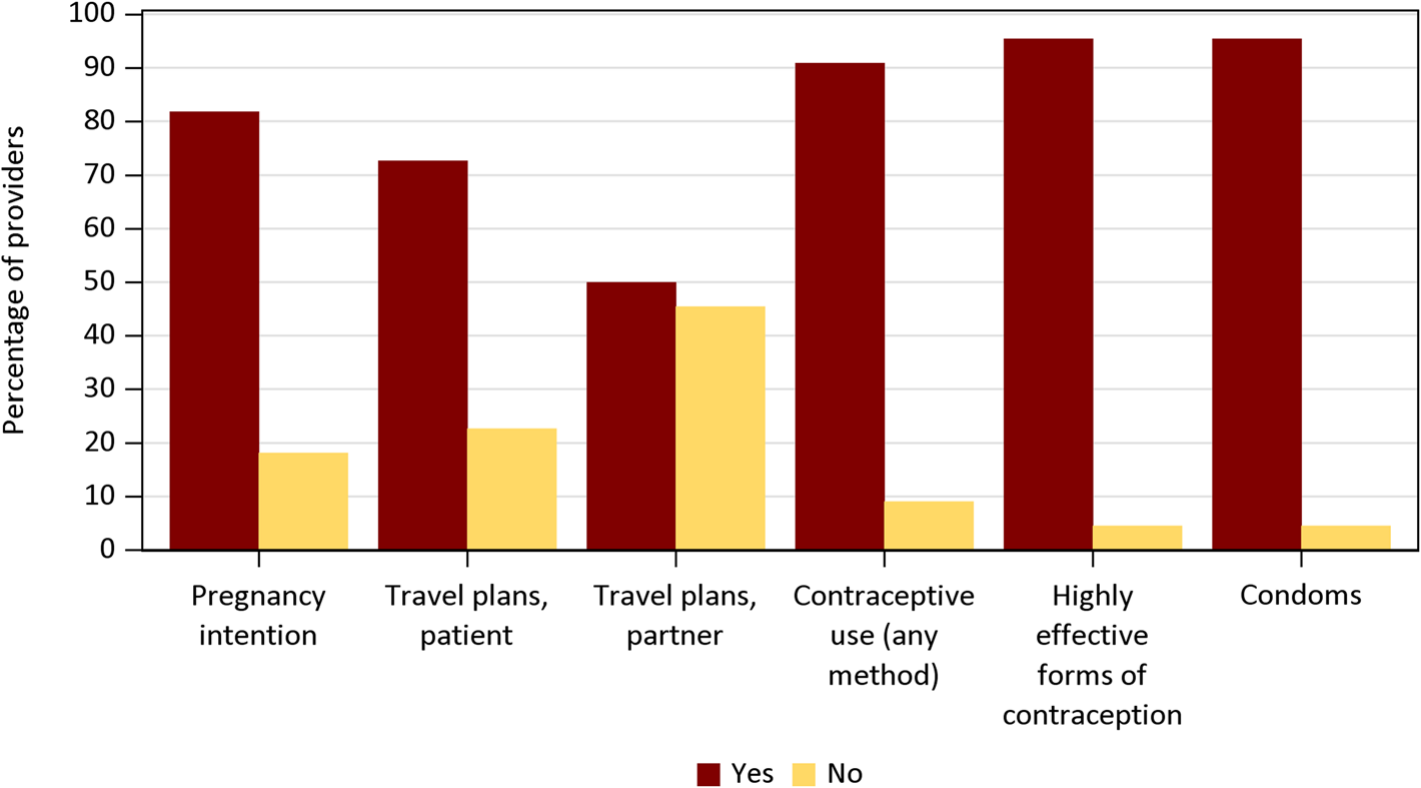
Adherence to CDC ZIKV counseling guidelines for non-pregnant women. Percentage of providers following specific CDC ZIKV counseling guidelines when asked “In your current practice, do you counsel non-pregnant women of reproductive age that you believe to be at risk of Zika on any of the following topics?” Each response on the y-axis is part of the CDC ZIKV guideline for preconception counseling and prevention of sexual transmission [6]. A “yes” answer indicates adherence to the guideline. *N* = 22.

On contraceptive and ZIKV knowledge questions, they averaged 69 and 81% correct respectively. Providers self-reported being slightly familiar (46%) or somewhat familiar (41%) with the CDC ZIKV guidelines for non-pregnant women, and most (59%) referenced these guidelines at least once a year. When posed with a hypothetical scenario of an at-risk patient, provider adherence to CDC ZIKV guidelines ranged from 50 to 95% (Fig. 3).

### Qualitative interview results

We interviewed, transcribed and analyzed transcripts from 15 providers. Eleven are physicians and 4 are APCs. Major themes corresponded to three domains of the Health Belief Model – Individual Perceptions, Modifying Factors, and Likelihood of Action (Fig. 1).

#### Individual perceptions

The major themes arising in this domain included (1) conditional risk and (2) prioritization of risk by pregnancy intention. Providers explained the risk of ZIKV to their non-pregnant patients as conditional on the timing of pregnancy and possible ZIKV exposure, the travel plans of the patient or partner to endemic areas, and the possibility of ZIKV transmission either vertically or sexually. Participants prioritized the threat of ZIKV to women intending pregnancy over those at risk for an unintended pregnancy or actively avoiding pregnancy, even though they acknowledge the fluidity of pregnancy intention and risk of unintended pregnancy: “[T] he only difference between a pregnant woman and a woman who’s currently sexually active and may get pregnant is just timing” (33 year old man, APC). However, if the conditions of timing or travel were not met, providers did not see the need to include ZIKV in contraceptive counseling: “If they want to get pregnant, then [Zika] will probably come up in the conversation. But if they’re not planning to get pregnant and are using birth control, I probably would not take an extra five minutes to tell them about Zika” (33 year old woman, APC).

#### Modifying factors

Modifying factors qualified the perceived threat of ZIKV and further weakened the risk of ZIKV to non-pregnant women. Major themes under this domain included (1) use of CDC ZIKV guidelines, (2) presence of ZIKV in the media, and (3) prior clinical experiences involving ZIKV. Participants identified several cues to action for prompting ZIKV-aware contraceptive counseling including: access to and use of CDC ZIKV guidelines, the distribution of those guidelines and other resources through provider meetings and email list serves, the presence or absence of ZIKV in the media and its influence on patients and providers, and the effect that caring for a pregnant patient with a positive ZIKV had on their practice. However, these cues went largely unapplied to the non-pregnant patient population. As one provider stated in regards to the non-pregnant CDC ZIKV guidelines: “I believe that they would probably be useful if I was looking at them. But I’ll be honest with you, I just haven’t” (39 year old, woman, APC).

#### Likelihood of action

The third piece in the Health Belief Model describes the likelihood of performing ZIKV-aware contraceptive counseling. No providers mentioned a perceived benefit of including ZIKV in routine contraceptive counseling. Provider factors such as comfort level and perceived self-efficacy in counseling Hispanic/Latina women about contraception did not arise as a barrier to contraceptive counseling. Two major themes of barriers included (1) prioritization of other health risks over ZIKV: “It’s [ZIKV] definitely important and it’s a public health threat, but where does it fall in my priority level, it just ends up being less than prenatal vitamins and weight loss counseling” (34 year old, woman, physician); and (2) the relative unimportance of patients’ risk of unintended pregnancy: “I mean, the question is do I universally recommend [contraception to] all my Latino [sic] women because they might be at risk of traveling without telling me? Do I urge contraception with the discussion of Zika involved? I don’t” (52 year old, man, physician). As a result, providers didn’t see the need to change their contraceptive counseling to include a discussion of ZIKV for women not seeking pregnancy.

## Discussion

Our mixed methods results indicate that PCPs are knowledgeable about ZIKV and its risks, CDC ZIKV guidelines, and contraception. However, the general consensus across providers in our qualitative data did not support incorporation of ZIKV risk assessment into contraceptive counseling for non-pregnant women. Our chart review data did not find a significant increase in either documentation of contraceptive counseling or ZIKV risk assessment during the pandemic and our qualitative analysis revealed several conditional and modifying factors that restricted uptake of ZIKV-aware contraceptive counseling. Prior work demonstrates stated pregnancy intention is not always predictive of behavior [19], and rates of pregnancy categorized as unintended or mistimed among the Hispanic/Latinx patient population is high (50%) [10]. These factors and our results point to an unmet need for ZIKV-aware contraceptive counseling in at-risk populations.

Although we observed a non-significant increase in contraceptive counseling among Hispanic/Latinx patients during the intervention period, it is difficult to ascertain the origin of this trend. Given the low rates of documentation of ZIKV risk assessment and the lack of ZIKV-aware contraceptive counseling evidenced by the qualitative portion, it is unlikely this trend is due to the ZIKV pandemic. Additionally, Utah did not experience endemic transmission of ZIKV and had fewer cases than other states. This makes it likely for ZIKV to be a lower priority. However, pregnant patients from the CHC comprised half of positive ZIKV tests in the state of Utah (52 total positive tests, 26 in pregnant women, Jan 2015 through June 2019) (H. Rettler, Utah Department of Health, personal communication, July 21, 2020) and many providers expressed concern over ZIKV in their specific patient population.

Previous studies on provider ZIKV knowledge support our findings that providers are knowledgeable about ZIKV [20]. Our participants scored similarly on contraceptive knowledge questions unrelated to ZIKV as in a prior study of obstetrician/gynecologist and family medicine physicians [16]. Although participants demonstrated a high level of CDC ZIKV guideline adherence when posed with a hypothetical non-pregnant patient at risk of ZIKV, this was not borne out in their current practice as demonstrated by the qualitative results.

A significant limitation of the chart review is relying on documentation of counseling or ZIKV risk assessment in determining actual practice. It is possible that contraceptive counseling and ZIKV risk assessment rates did change during the ZIKV pandemic, however the provider interviews support minimal change in counseling rate during this time. We did not collect information on pregnancy intention from the chart review nor could we determine contraceptive counseling need. This may account for the lack of change in contraceptive counseling and overall low rate of counseling (40%). Although prior studies of documented contraceptive counseling rates among PCPs are lacking, documentation of contraceptive counseling ranges from 19 to 35% in specialty clinics [21, 22]. Limitations in the provider portion of this study are those inherent to a descriptive study design, including a small number of participants and limited generalizability. This limits external validity. Additionally, limitations of the ITSA framework include the assumption of a pre-existing linear trend in Zika Assessment that would continue without the intervention (CDC guidance), and the lack of control patient data to compare changes following the guidance. The strengths of this work include its large number of data points for the ITSA and its timeliness for assessing the effect on contraceptive counseling of an emerging pathogen with vertical transmission and achieving thematic saturation in the qualitative portion.

## Conclusions

Primary care providers are the first line in helping patients navigate their reproductive life plans when novel and emerging pathogens arise. Our findings stress the importance of developing methods to better translate knowledge, risk perception, and guidelines into easily actionable practices to improve contraceptive counseling in busy primary care settings. As one participant said, ZIKV is a “disease du jour” and there will be more emerging diseases that affect women of reproductive age.

## Supplementary Information

**Additional file 1.**

## Data Availability

The datasets used and/or analyzed during the current study are available from the corresponding author on reasonable request.

## Abbreviations

ZIKV: Zika virus
CDC: Centers for Disease Control and Prevention
CHC: Community Health Centers, Inc.
NP: Nurse practitioner
PA: Physician assistant
ITSA: Interrupted time series analysis
PCP: Primary care providers
APC: Advanced practice clinicians
GLMM: Generalized linear mixed effects model
