# Supplementary material for: Determining the impact of the Zika pandemic on primary care providers’ contraceptive counseling of non-pregnant patients in the US: a mixed methods study
Source: BMC Health Serv Res. 2021 Nov 9;21:1215. doi: 10.1186/s12913-021-07170-0 (PMC8579600; doi:10.1186/s12913-021-07170-0)
Supplement: Supplementary file 1 — Additional file 1. [file 12913_2021_7170_MOESM1_ESM.pdf]

Additional file 1: Zika and Contraceptive knowledge and practice survey instrument.

PDF format. This file contains the survey instrument distributed to participants to assess Zika and contraceptive knowledge and practices. Questions were created from existing published knowledge questions, CDC Zika guidelines, and de novo to address study aims.

Contraception and Zika Virus

Thank you for agreeing to participate in our study about contraceptive counseling and Zika. The survey portion will take approximately 10 minutes to complete. Two of the sections below ask specifics about your practice regarding contraception and Zika. Please answer these questions to the best of your ability based on your understanding of the topics.

How many years have you practiced at the Community Health Centers (CHCs)? ______________________________

In the average half-day of clinic, how many women of reproductive age, excluding pregnant women, do you see? ______________________________

Do you provide reproductive health counseling in your current practice with the CHCs (i.e. preconception counseling, contraceptive counseling, STD counseling, etc)?

◯ Yes
◯ No

For each of the following types of contraception, indicate which of the following you may discuss during a contraceptive visit.

	Yes	No
Hormonal IUD (Mirena, Skyla, Kyleena, Liletta)	◯	◯
Non-hormonal IUD (Paragard)	◯	◯
Contraceptive implant (Nexplanon)	◯	◯
Combined oral contraceptives	◯	◯
Female sterilization	◯	◯
Male sterilization	◯	◯
Progestin injection (Depo)	◯	◯
Contraceptive patch	◯	◯
Vaginal ring (Nuvaring)	◯	◯
Progestin only pill	◯	◯
Condoms	◯	◯
Diaphragm	◯	◯
Cervical cap	◯	◯
Spermicide	◯	◯
Fertility awareness based method or natural family planning	◯	◯
Emergency contraception	◯	◯

For each of the following types of contraception, indicate whether or not you provide or prescribe them to a patient should she be an appropriate candidate and desire that method.

	Yes	No
Hormonal IUD (Mirena, Skyla, Kyleena, Liletta)	◯	◯
Non-hormonal IUD (Paragard)	◯	◯
Contraceptive implant (Nexplanon)	◯	◯
Combined oral contraceptives	◯	◯
Female sterilization	◯	◯
Male sterilization	◯	◯
Progestin injection (Depo)	◯	◯
Contraceptive patch	◯	◯
Vaginal ring (Nuvaring)	◯	◯
Progestin only pill	◯	◯
Condoms	◯	◯
Diaphragm	◯	◯
Cervical cap	◯	◯
Spermicide	◯	◯
Fertility awareness based method or natural family planning	◯	◯
Emergency contraception	◯	◯

Do you insert hormonal IUDs (Mirena, Liletta, etc)?

◯ Yes
◯ No

Which types of hormonal IUDs do you insert?

◯ Only Mirena/Skyla/Kyleena
◯ Only Liletta
◯ All hormonal IUD types

Do you insert non-hormonal IUDs (Paragard)?

◯ Yes
◯ No

Do you insert hormonal implants (Nexplanon)?

◯ Yes
◯ No

Does a patient's access to medical insurance influence your contraception prescribing practices in clinic?

◯ Yes
◯ No

In what way does a patient's access to medical insurance influence your contraceptive prescribing practices in clinic?

☐ It influences the type of contraception I prescribe
☐ It influences the brand of contraception I prescribe
☐ It influences my decision to refer to another clinic or organization for an IUD/subdermal implant
☐ It influences the contraception options I present to the patient

Next, we'd like to ask you some questions about contraceptives. Please answer each to the best of your ability.

How much does the use of intrauterine contraception increase a woman's risk of infertility?

- ◯ Definitely doesn't increase
- ◯ Probably doesn't increase
- ◯ Probably does increase
- ◯ Definitely does increase
- ◯ Unsure

Compared to women not using an IUD, to what extent are women using an IUD at increased risk for pelvic inflammatory disease?

- ◯ Definitely not at increased risk
- ◯ Probably not at increased risk
- ◯ Probably at increased risk
- ◯ Definitely at increased risk
- ◯ Unsure

How unlikely or likely are you to prescribe combined hormonal contraceptives to women with migraine with aura exclusively for contraceptive purposes?

- ◯ Very unlikely
- ◯ Somewhat unlikely
- ◯ Somewhat likely
- ◯ Very likely
- ◯ Unsure

How unlikely or likely are you to prescribe progestin-only contraceptives to women with a history of deep venous thrombosis or pulmonary embolism?

- ◯ Very unlikely
- ◯ Somewhat unlikely
- ◯ Somewhat likely
- ◯ Very likely
- ◯ Unsure

If a woman has hypertension, even if well controlled, how unlikely or likely are you to prescribe her combined hormonal contraception?

- ◯ Very unlikely
- ◯ Somewhat unlikely
- ◯ Somewhat likely
- ◯ Very likely
- ◯ Unsure

Please mark the contraceptive methods you believe have the highest efficacy with typical use (< 1% failure rate) . Please select all that apply

- ☐ Hormonal IUD
- ☐ Non-hormonal IUD
- ☐ Implant
- ☐ Combined oral contraceptives
- ☐ Female sterilization
- ☐ Male sterilization
- ☐ Progestin injection
- ☐ Patch
- ☐ Vaginal ring
- ☐ Progestin only pill
- ☐ Condoms
- ☐ Diaphragm
- ☐ Cervical cap
- ☐ Spermicide
- ☐ Fertility awareness based method or natural family planning

In the following section, we'd like to ask you questions about Zika virus, its prevention, and its effects on pregnancy. We understand some of these questions may be difficult or you may not know. Please answer to the best of your ability.

As best you know, has mosquito transmission of Zika been confirmed in the continental United States?

◯ Yes
◯ No
◯ Unsure

To the best of your knowledge, is it possible to contract Zika by mosquito transmission in Utah currently?

◯ Yes
◯ No
◯ Unsure

As far as you know, how can a person contract Zika?

	Yes	No	Unsure
From an infected mosquito bite	◯	◯	◯
From drinking or washing in polluted water	◯	◯	◯
From unprotected sexual intercourse	◯	◯	◯
From air borne transmission	◯	◯	◯
From breast milk	◯	◯	◯
From a blood transfusion	◯	◯	◯
From mother to child transmission	◯	◯	◯
Other	◯	◯	◯

If other, please specify

____

To the best of your knowledge, what are the signs and symptoms of acute Zika infection?

	Yes	No	Unsure
Fever	◯	◯	◯
Headache	◯	◯	◯
Rash	◯	◯	◯
Joint pain	◯	◯	◯
Conjunctivitis	◯	◯	◯
Diarrhea	◯	◯	◯

Does everybody who contracts Zika show symptoms?

◯ Yes
◯ No
◯ Unsure

According to what you know, how can a patient prevent Zika infection?

	Yes	No	Unsure
Practice mosquito avoidance like using a mosquito net, repellent, and wearing covering clothing	◯	◯	◯
Use a condom in all sexual relations	◯	◯	◯
Use other forms of contraception	◯	◯	◯
Abstain from sexual intercourse	◯	◯	◯

To the best of your knowledge, which of the following findings comprise Congenital Zika Syndrome?

- ☐ Severe microcephaly
- ☐ Decreased brain tissue with a specific pattern of brain damage
- ☐ Damage to the back of the eye
- ☐ Joints with limited range of motion
- ☐ Increased muscle tone
- ☐ All of the above

Beginning in 2016, the CDC began issuing Zika counseling and practice guidelines for providers caring for women of reproductive age. In the following section, we'd like to ask about your familiarity with and use of the CDC Zika guidelines.

How familiar are you with the CDC Zika guidelines for non-pregnant women of reproductive age?

- ◯ Not familiar at all
- ◯ Slightly familiar
- ◯ Somewhat familiar
- ◯ Very familiar

How often have you referenced the CDC Zika guidelines for non-pregnant women in your clinical practice over the past year?

- ◯ Once a day
- ◯ At least once a week
- ◯ At least once a month
- ◯ As least once a year
- ◯ Never

In your current practice, do you counsel non-pregnant women of reproductive age that you believe to be at risk of Zika on any of the following topics?

	Yes	No
Pregnancy intention in the next year	◯	◯
Travel plans of the women	◯	◯
Travel plans of the woman's partner	◯	◯

If a woman who you believe to be at risk for Zika intends to become pregnant, do you discuss any of the following counseling topics?

	Yes	No

If the woman has possible Zika exposure, she should abstain from sexual intercourse for 8 weeks from last possible exposure	○	○
If the woman has possible Zika exposure, the woman's male partner should use condoms for 8 weeks from last possible exposure	○	○
If the woman's male partner has possible Zika exposure, the couple should abstain from sexual intercourse for 6 months from last possible exposure	○	○
If the woman's male partner has possible Zika exposure, the couple should use condoms for 6 months from last possible exposure	○	○
If the woman's male partner has possible Zika exposure, the couple should abstain from sexual intercourse for 3 months from last possible exposure	○	○
If the woman's male partner has possible Zika exposure, the couple should use condoms for 3 months from last possible exposure	○	○

If a woman who you believe to be at risk for Zika does not intend to become pregnant in the next year, do you recommend any of the following?

	Yes	No
Contraceptive use in general (any method)	○	○
Use of highly effective forms of contraception (IUDs, implant, sterilization)	○	○
Use of condoms	○	○

Finally, please provide us with the following demographic information.

What is your age?	__________________________________
What is your gender?	◯ Man<br>◯ Woman<br>◯ Prefer not to answer<br>◯ Other
If other, please specify.	__________________________________
How would you classify your race?	◯ White<br>◯ Black or African America<br>◯ Asian<br>◯ American Indian<br>◯ Pacific Islander<br>◯ Other
If other, please specify	__________________________________
How would you classify your ethnicity?	◯ Latino/a or Hispanic<br>◯ Not Latino/a or Hispanic
Please select your provider (certification) type.	◯ MD<br>◯ DO<br>◯ PA<br>◯ NP
How many years has it been since you completed medical training (i.e. completion of residency, PA/NP school)?	__________________________________
Since completing training, how many years total have you practiced in primary care?	__________________________________
Since completing training, how many years total have you cared for Hispanic/Latino patients?	__________________________________
Please enter your email address to receive the gift card.	__________________________________
